# Subgroup analysis of phase 2 study of ceftolozane/tazobactam in neonates and young infants with pyelonephritis

**DOI:** 10.1128/spectrum.01800-23

**Published:** 2023-09-12

**Authors:** Emmanuel Roilides, John S. Bradley, Julia Lonchar, Jennifer A. Huntington, Prachi Wickremasingha, Feng-Hsiu Su, Christopher J. Bruno, Matthew G. Johnson

**Affiliations:** 1 Third Department of Pediatrics, Infectious Diseases Unit, School of Medicine, Aristotle University and Hippokration General Hospital, Thessaloniki, Greece; 2 Department of Pediatrics, University of California, San Diego School of Medicine and Rady Children’s Hospital of San Diego, San Diego, California, USA; 3 Merck & Co., Inc., Rahway, New Jersey, USA; Mayo Foundation for Medical Education and Research, Rochester, Minnesota, USA

**Keywords:** ceftolozane/tazobactam, neonates, cUTI, young infants, pyelonephritis

## Abstract

Ceftolozane/tazobactam is approved for the treatment of patients from birth to <18 y old with complicated urinary tract infections (cUTI). This post hoc analysis evaluated the safety, efficacy, and pharmacokinetics (PK) of ceftolozane/tazobactam compared with meropenem in neonates and young infants. NCT03230838 was a phase 2, randomized, active comparator-controlled, double-blind study of patients from birth to <18 y of age with cUTI, including pyelonephritis, given ceftolozane/tazobactam or meropenem in a 3:1 ratio. This subset analysis included only neonates and young infants < 3 mo of age. The microbiologic modified intent-to-treat population (mMITT) included 20 patients (ceftolozane/tazobactam, *n* = 14; meropenem, *n* = 6). All patients had pyelonephritis at baseline; two patients in each treatment group had bacteremia (overall 4/20, 20%). *Escherichia coli* was the most common baseline pathogen (overall 16/20, 80%). Safety and efficacy results were similar between treatment groups and consistent with the overall pediatric population. There were no serious drug-related adverse events (AEs), no discontinuations due to AEs, and no AEs leading to death in either treatment group. For the ceftolozane/tazobactam and meropenem treatment groups, clinical cure rates in the mMITT population were 92.9% and 100%, respectively. The population PK analysis of neonates and young infants demonstrated similar ceftolozane and tazobactam exposures to those of adults, achieving pharmacodynamic targets associated with clinical and microbiologic cure. Ceftolozane/tazobactam has a favorable safety profile and achieves high clinical cure and microbiologic eradication rates in neonates and young infants < 3 mo of age with cUTI and pyelonephritis.

**Importance:**

Extrapolation of antibacterial agent pharmacokinetics from adults to newborns and young infants may not be appropriate; similarly, the clinical manifestations of infectious diseases and outcomes following antibacterial treatment may not be similar. Ceftolozane/tazobactam is an antibacterial drug combination active against *Pseudomonas aeruginosa* and other multidrug-resistant gram-negative bacteria. A clinical study led to the approval for ceftolozane/tazobactam in patients from birth to 18 y of age who have complicated urinary tract infections, including those with serious kidney infections. Based on data collected during that clinical study, we compared newborns and young infants who were treated with ceftolozane/tazobactam (14 patients) and those who were treated with meropenem (6 patients). We found that ceftolozane/tazobactam treatment of newborns and young infants up to 3 mo of age who have complicated urinary tract infections demonstrated a favorable safety profile and high clinical cure and microbiologic eradication rates, similar to meropenem.

## INTRODUCTION

Urinary tract infection (UTI) is one of the most common bacterial infections in infants, with prevalence rates among the 0 to 3 mo age group of up to 20% in uncircumcised males and up to 13% in females, and it is commonly associated with congenital anomalies of the kidney and urinary tract (1, 2). Higher rates of recurrent UTI have been associated with infants aged <6 mo (3, 4) compared with older children. Bacteremia has been observed to be more common in patients who are <3 mo of age with UTIs (5).

The treatment of multidrug-resistant infections in neonates and young infants is an unmet medical need. *Escherichia coli* is one of the pathogens most commonly associated with neonatal sepsis, followed by *Klebsiella pneumoniae*. These pathogens are also the most common causative pathogens (80% and 16%, respectively) of pediatric UTI and are increasingly characterized as being resistant to antibacterial agents (6, 7). Extended-spectrum β-lactamase (ESBL)-producing Enterobacterales has an estimated global pooled prevalence of 14% among pediatric UTI cases (8); in the United States, the frequency of ESBL-producing *E. coli* in community-onset bacteriuria episodes increased from 0.97% in 2015 to 3.54% in 2020 (9). In the United States, between 1999 and 2011, 74% of ESBL-producing Enterobacterales and 20% of *Pseudomonas aeruginosa* isolates from pediatric patients were multidrug resistant (10, 11). These resistance rates underscore the need for new treatment options for multidrug-resistant infections in neonates and young infants.

Currently, there is a lack of clinical data for antibacterial treatments for neonates and young infants owing to their historical exclusion from clinical studies (12) and great difficulties in conducting studies in this age range. For example, challenges include obtaining parental consent for an experimental intervention in neonates and the historic use of laboratory assays that required larger blood samples compared with current assays (13, 14). This has resulted in the common use of drugs (~65% overall) that are off-label or unlicensed in this population, despite an increasing awareness that the pharmacokinetic (PK) parameters of neonates and young infants differ from adults and older children because of the rapid physiologic changes and population-specific pathophysiology during the first 3 mo of life (12, 15 – 17). Based on legislation, regulatory agencies require new drugs to be studied in newborns unless these studies are deferred or waived; however, data for neonatal and young infant populations remain sparse owing to the challenges encountered in enrolling this population (18). Consequently, off-label drug use rates in neonatal intensive care units have been reported to be as high as 51% (19, 20).

Ceftolozane/tazobactam, a cephalosporin–β-lactamase inhibitor combination, is approved to treat complicated UTI (cUTI), including pyelonephritis, complicated intra-abdominal infections (cIAI), and nosocomial pneumonia in adults (21, 22). We have completed three clinical studies of ceftolozane/tazobactam in children from birth to 18 y of age: a PK and safety study enrolling patients with proven or suspected gram-negative infection receiving a single intravenous (IV) dose (23) and two phase 2 studies, one evaluating the safety and efficacy in pediatric patients with cUTI, including pyelonephritis (24), and another in pediatric patients with cIAI (25). In both phase 2 studies, ceftolozane/tazobactam had a favorable safety profile and achieved high clinical cure and microbiologic eradication rates. Together, these studies formed the basis for the pediatric approval of ceftolozane/tazobactam for use in the treatment of cUTI (including pyelonephritis) and cIAI in the United States and the European Union (22, 26).

Here, we describe an exploratory post hoc analysis evaluating the PK, safety, and efficacy of ceftolozane/tazobactam compared with meropenem in neonates and young infants who were included in the phase 2 study of pediatric patients with cUTI. Neonates are recognized as a unique population from the perspective of PK (given the ontogeny of biologic processes relevant to drug disposition) and from the perspective of safety (given the relative immaturity of organ function during the first weeks of life) and efficacy (given that neonates are immune deficient compared with older children and adults). Therefore, details of PK, safety, and efficacy in neonates and young infants are presented separately and in more detail than those recently published for all children (21).

## MATERIALS AND METHODS

NCT03230838 (protocol MK-7625A-034) was a phase 2, randomized, active comparator-controlled, double-blind study of patients from birth to <18 y of age with cUTI, including pyelonephritis. This analysis of neonates (birth to <28 d) and young infants (28 d to <3 mo) includes only those patients from birth (defined as >32 wk of gestational age and ≥7 d postnatal) to <3 mo of age. The pediatric dosing recommendations for ceftolozane/tazobactam were developed using data from patients from birth (>32 wk of gestational age and ≥7 d postnatal) to <18 y of age; no data were available at that time from patients ≤32 wk of gestational age or <7 d postnatal age (27). Therefore, this analysis assessed those infants >32 wk of gestational age and ≥7 d postnatal age.

Patients who met any of the following criteria at randomization were excluded: a history of cUTI caused by a pathogen known to be resistant to either IV study treatment; receipt of potentially therapeutic antibacterial therapy for >24 h during the 48 h preceding the first dose of study treatment (except in cases of patients receiving >48 h of prior antibacterial therapy that were deemed clinical or microbiologic failures); or an estimated glomerular filtration rate (eGFR) <50 mL/min/1.73 m^2^ (28). Patients were given ceftolozane 20 mg/kg and tazobactam 10 mg/kg; those in the meropenem group were given 20 mg/kg, with higher dosing up to 30 mg/kg for patients who were 14 d to <3 mo of age (29) permitted at the investigator’s discretion. Each dose of ceftolozane/tazobactam or meropenem was administered as a 60-min (±10 min) infusion every 8 h (±1 h). After receiving at least nine doses of double-blind IV study treatment, patients could be switched to open-label, standard-of-care, oral step-down therapy at the investigator’s discretion. The total duration of study treatment (IV only or IV + oral) was a minimum of 7 d and a maximum of 14 d, but low-dose prophylaxis was allowed after completion of study treatment if clinically indicated. Details of the study design, patient eligibility and exclusion criteria, treatment, specimen collection, and statistical analysis have been previously described (24).

The primary objective was to evaluate the safety and tolerability of ceftolozane/tazobactam compared with meropenem. Primary end points were determined in the safety population, consisting of all randomized patients who received any amount of study treatment, and included adverse events (AE) and changes in laboratory values or vital signs through the last follow-up visit (28–35 d after the last dose). Secondary end points were clinical success rate (defined as cure) and per-patient microbiologic eradication at the end-of-treatment (EOT) and test-of-cure (TOC) visits in the microbiologic modified intent-to-treat (mMITT) population, which included all randomized patients who received any amount of study treatment and had ≥1 causative uropathogen from a study-qualifying baseline urine culture. For patients who received oral step-down therapy, the EOT visit was scheduled <48 h after the last oral dose; if no oral step-down therapy was given, the end of IV treatment visit (<24 h after the last IV dose) served as the EOT visit. For all patients, the TOC visit occurred 5–9 d after the last dose of study treatment (IV study treatment or oral step-down therapy, if applicable). Three blood samples were collected from patients on day 3 of dosing (after receiving at least 6 doses of treatment) and plasma concentrations of ceftolozane and tazobactam were determined and assessed in the context of the Clinical and Laboratory Standards Institute (CLSI) and the European Committee on Antimicrobial Susceptibility Testing (EUCAST) breakpoints for *P. aeruginosa* and Enterobacterales (30, 31). These blood samples were collected at the following three time points: at the end of infusion (1.0 h after the start of infusion of IV study treatment, within 10 min after the end of total dose administration); between 4.0 and 5.0 h after the start of infusion; and between 7.0 and 8.0 h after the start of infusion but before the start of the next dose of study treatment. Provisions were included in the protocol to allow for sample collection at comparable time points after day three in patients who continued to receive IV study treatment, and sample collection was not required over the same dosing interval.

Plasma ceftolozane and tazobactam concentrations were determined using a validated high-performance liquid chromatographic tandem mass spectrometric method by Pharma Medica Research Inc. (Mississauga, ON, Canada). The lower limit of quantification was 0.25 and 0.10 µg/mL for ceftolozane and tazobactam, respectively. The analytical ranges were 0.25–150.0 and 0.10–50.0 µg/mL for ceftolozane and tazobactam, respectively. The PK data were included in population PK analysis using the software packages Perl-Speaks-NONMEM 5.0.0 and NONMEM version 7.4.4 (ICON plc).

## RESULTS

### Patients included in the subgroup analysis

The mMITT population included a total of 20 children < 3 mo of age (3 neonates and 17 young infants); 14 in the ceftolozane/tazobactam group and 6 in the meropenem group. Patients were enrolled at study sites in Greece (13 patients at four sites), Hungary (5 patients at two sites), and the United States (2 patients at one site). The baseline characteristics of patients were comparable between treatment groups (Table 1). All patients had pyelonephritis at baseline (diagnosis at the discretion of the investigator), although three patients had urological abnormalities as well (one patient with a history of hydronephrosis, decreased renal function, and pyeloureteral junction stenosis; one patient with vesicoureteral reflux; and one patient with unspecified congenital/anatomic abnormalities). Bacteremia was present in two patients in each treatment group (ceftolozane/tazobactam, 14.3%; meropenem, 33.3%). Urine samples were collected at baseline by catheter in nine (64.3%) patients in the ceftolozane/tazobactam group and in five (83.3%) patients in the meropenem group, by midstream clean catch in one (7.1%) patient in the ceftolozane/tazobactam group and by suprapubic aspiration in four (28.6%) patients in the ceftolozane/tazobactam group and one (16.7%) patient in the meropenem group.

**TABLE 1:** Demographics and baseline characteristics for neonates and young infants (mMITT population)^*a*^

Characteristic	C/T (*n* = 14)	MEM (*n* = 6)
Male, *n* (%)	12 (85.7)	4 (66.7)
Median (range) age, d	37.6 (23.0–78.1)	51.1 (9.9–86.1)
White race, *n* (%)	14 (100)	6 (100)
Median (range) weight, kg	4.5 (2.6–6.2)	4.8 (3.8–5.7)
Baseline pyelonephritis diagnosis, *n* (%)	14 (100)	6 (100)
Bacteremia at baseline, *n* (%)	2 (14.3)	2 (33.3)
Baseline eGFR (mL/min/1.73 m^2^),^*b*^ *n* (%)
eGFR ≥80	4 (28.6)	1 (16.7)
eGFR ≥50 to <80	10 (71.4)	4 (66.7)
eGFR ≥30 to <50	0	1 (16.7)
Method of baseline urine sample collection, *n* (%)
Urinary catheter	9 (64.3)	5 (83.3)
Suprapubic aspiration	4 (28.6)	1 (16.7)
Midstream clean catch	1 (7.1)	0
Qualifying baseline uropathogens, *n* (%)
*Escherichia coli*	11 (78.6)	5 (83.3)
*Klebsiella pneumoniae*	2 (14.3)	0
*Pseudomonas aeruginosa*	0	1 (16.7)
*Citrobacter* spp.	1 (7.1)	0
Failure of prior antibacterial therapies, *n* (%)	0	0

^*a*^ C/T, ceftolozane/tazobactam; eGFR, estimated glomerular filtration rate; MEM, meropenem; mMITT, microbiologic modified intent-to-treat.

^*b*^ eGFR was calculated using the revised Bedside Schwartz equation (28) at baseline.

### Pathogens at baseline

*E. coli* was the most common qualifying baseline pathogen in the mMITT group [ceftolozane/tazobactam, 11 (78.6%); meropenem, 5 (83.3%)]. *K. pneumoniae* and *Citrobacter* spp. were isolated from two (14.3%) and one (7.1%) patients, respectively, in the ceftolozane/tazobactam group; *P. aeruginosa* was isolated from one patient in the meropenem group. No ESBL-producing or multidrug-resistant pathogens were isolated. The minimum inhibitory concentration (MIC) values of the baseline pathogens isolated are presented in Table 2.

**TABLE 2:** MIC values for baseline pathogens^*a*^

	C/T			MEM		
	MIC_50_ (µg/mL)	MIC_90_ (µg/mL)	MIC range (µg/mL)	MIC_50_ (µg/mL)	MIC_90_ (µg/mL)	MIC range (µg/mL)
Enterobacterales (*n* = 18)	0.12	0.25	0.12–1.0	≤0.06	≤0.06	≤0.06–≤0.06
*E. coli* (*n* = 15)^*b*^	0.12	0.25	0.12–0.25	≤0.06	≤0.06	≤0.06–≤0.06
*K. pneumoniae* (*n* = 2)	0.625	1.0	0.25–1.0	≤0.06	≤0.06	≤0.06–≤0.06
*Citrobacter* spp. (*n* = 1)	0.25	0.25	n/a	≤0.06	≤0.06	n/a
*P. aeruginosa* (*n* = 1)	0.5	0.5	n/a	0.5	0.5	n/a

^*a*^ C/T, ceftolozane/tazobactam; MEM, meropenem; MIC, minimum inhibitory concentration; n/a, not applicable.

^*b*^ For one patient with baseline *E. coli* isolates, the specimen was not sent to the central microbiology laboratory, but the local microbiology laboratory reported a Kirby-Bauer result of 27 mm for ceftolozane/tazobactam (susceptible) and 30 mm for meropenem (susceptible).

### Safety

The mean (SD) duration of IV treatment was comparable in both treatment groups [ceftolozane/tazobactam, 5.9 (2.84) d; meropenem, 5.8 (2.72) d]. Overall mean (SD) treatment duration (both IV and oral stepdown) was comparable in both treatment groups [ceftolozane/tazobactam, 11.0 (2.72) d; meropenem, 11.6 (1.94) d]. A total of 12 (85.7%) and 5 (83.3%) patients switched to optional oral step-down therapy for a mean (SD) duration of 5.9 (1.27) d and 7.0 (1.74) d in the ceftolozane/tazobactam and meropenem groups, respectively. The choice of oral step-down therapy was determined at the investigator’s discretion based on local microbiology laboratory results.

The overall incidence of AEs was comparable between the ceftolozane/tazobactam and meropenem groups (Table 3). There was one serious AE of pyelonephritis (a subsequent episode, not the original episode of pyelonephritis) of severe intensity in the ceftolozane/tazobactam group, which was not related to study treatment [occurred on day 37 (32 d after the last dose)] and resolved on day 46 with treatment. There were no serious drug-related AEs, no discontinuations due to AEs, and no AEs leading to death in either treatment group. Three (20%) patients in the ceftolozane/tazobactam group had an AE of thrombocytosis and 3 (20%) had neutropenia-related AEs; no patients in the meropenem group had thrombocytosis or neutropenia-related AEs. The thrombocytosis cases were of mild intensity and were determined by the investigator to not be drug related, none were considered to be serious, and all cases had resolved or were resolving at the time of the last study visit. All of the neutropenia-related AEs were considered to be drug related: two were classified by the investigator as being of moderate intensity and had durations of 5.9 and 4.9 wk; the other was classified as being of severe intensity and had a duration of 6.6 wk. All cases were resolved at the time of the last study visit.

**TABLE 3:** AEs for neonates and young infants (all patients as treated population)^*a*^

Patients, *n* (%)	C/T	MEM
	(*n* = 15)	(*n* = 6)
≥1 AE	9 (60.0)	3 (50.0)
≥1 drug-related AE^*b*^	4 (26.7)	1 (16.7)
Serious AE^*c*^	1 (6.7)	0
Serious drug-related AE^*b*^	0	0
Death	0	0
Discontinuation due to AE	0	0
Discontinuation due to drug-related AE^*b*^	0	0
Discontinuation due to serious AE	0	0
Most commonly reported AEs^*d*^		
Thrombocytosis	3 (20.0)	0
Neutropenia	2 (13.3)	0
Most commonly reported drug-related AEs^*b*^ ^,*d*^		
Neutropenia	2 (13.3)	0

^*a*^ AE, adverse event; C/T, ceftolozane/tazobactam; MEM, meropenem.

^*b*^ Determined by the investigator to be related to the drug.

^*c*^ Serious AE was pyelonephritis occurring in one patient in the C/T group.

^*d*^ AEs that occurred in ≥2 patients in either treatment group.

### Efficacy

**Fig 1:**
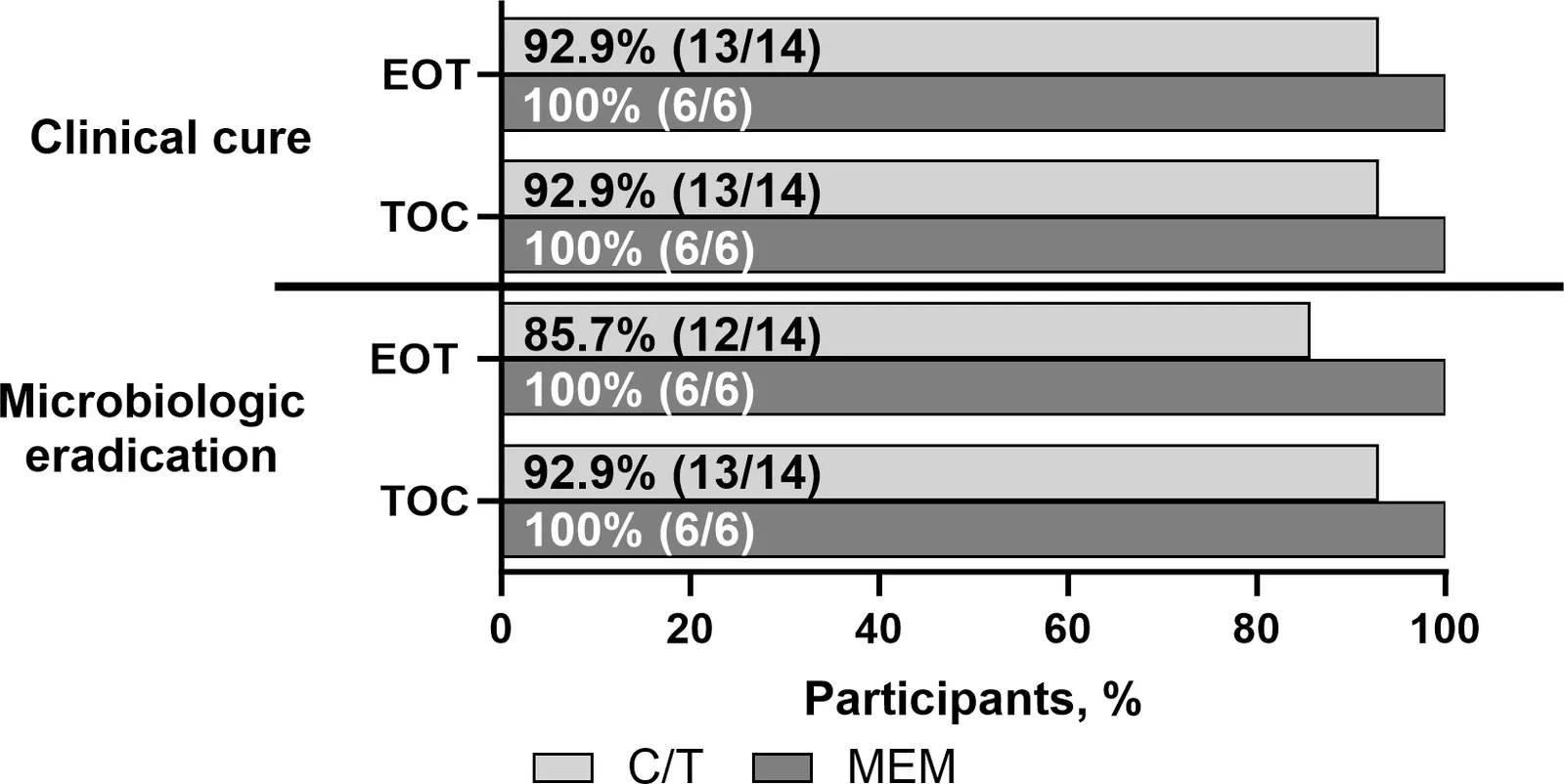
Clinical cure and microbiologic response at EOT and TOC for neonates and young infants (mMITT population). C/T, ceftolozane/tazobactam; EOT, end of treatment; MEM, meropenem; mMITT, microbiologic modified intent-to-treat; TOC, test of cure.

Clinical cure rates in the mMITT population were 92.9% and 100% for the ceftolozane/tazobactam and meropenem treatment groups, respectively, at both the EOT and TOC visits (Fig. 1). The one clinical failure in the ceftolozane/tazobactam arm occurred at the end of IV visit (EOIV) and was related to an obstruction of the urinary tract (posterior urethral valve) in a patient with pyelonephritis due to ceftolozane/tazobactam-susceptible *Citrobacter* spp. at baseline. This patient was considered a clinical failure per protocol because the study drug was stopped by the investigator and the patient’s IV antibacterial treatment was switched. However, this patient was not experiencing any new or persistent symptoms at the time the antibacterial therapy was switched, and follow-up urine cultures on therapy were sterile. Because of the clinical failure at EOIV, this patient was also considered a clinical failure at EOT and TOC per protocol.

Microbiologic eradication rates were 85.7% (ceftolozane/tazobactam) and 100% (meropenem) at the EOT visit and 92.9% (ceftolozane/tazobactam) and 100% (meropenem) at the TOC visit (Fig. 1). Two patients experienced microbiologic persistence at EOT; both were in the ceftolozane/tazobactam group. One patient in the ceftolozane/tazobactam arm experienced microbiologic persistence (with *E. coli*, susceptible to ceftolozane/tazobactam) at EOT and TOC and had an indwelling Foley catheter at the time of initial infection that was not subsequently changed. A second patient in the ceftolozane/tazobactam arm had presumed microbiologic persistence at EOT based on missing microbiologic data, but microbiologic eradication was achieved for this patient at TOC.

Bacteremia at baseline was detected in two patients in each treatment arm [14.3% (2/14) and 33.3% (2/6) in the ceftolozane/tazobactam and meropenem arms, respectively]. Clinical cure at EOT and TOC was achieved by one of two (50.0%) patients with bacteremia in the ceftolozane/tazobactam group (one patient with clinical failure as described previously with posterior urethral values) and by both (100%) patients with bacteremia in the meropenem group; microbiologic eradication was achieved by all patients with bacteremia at EOT and TOC.

### Pharmacokinetics

**Fig 2:**
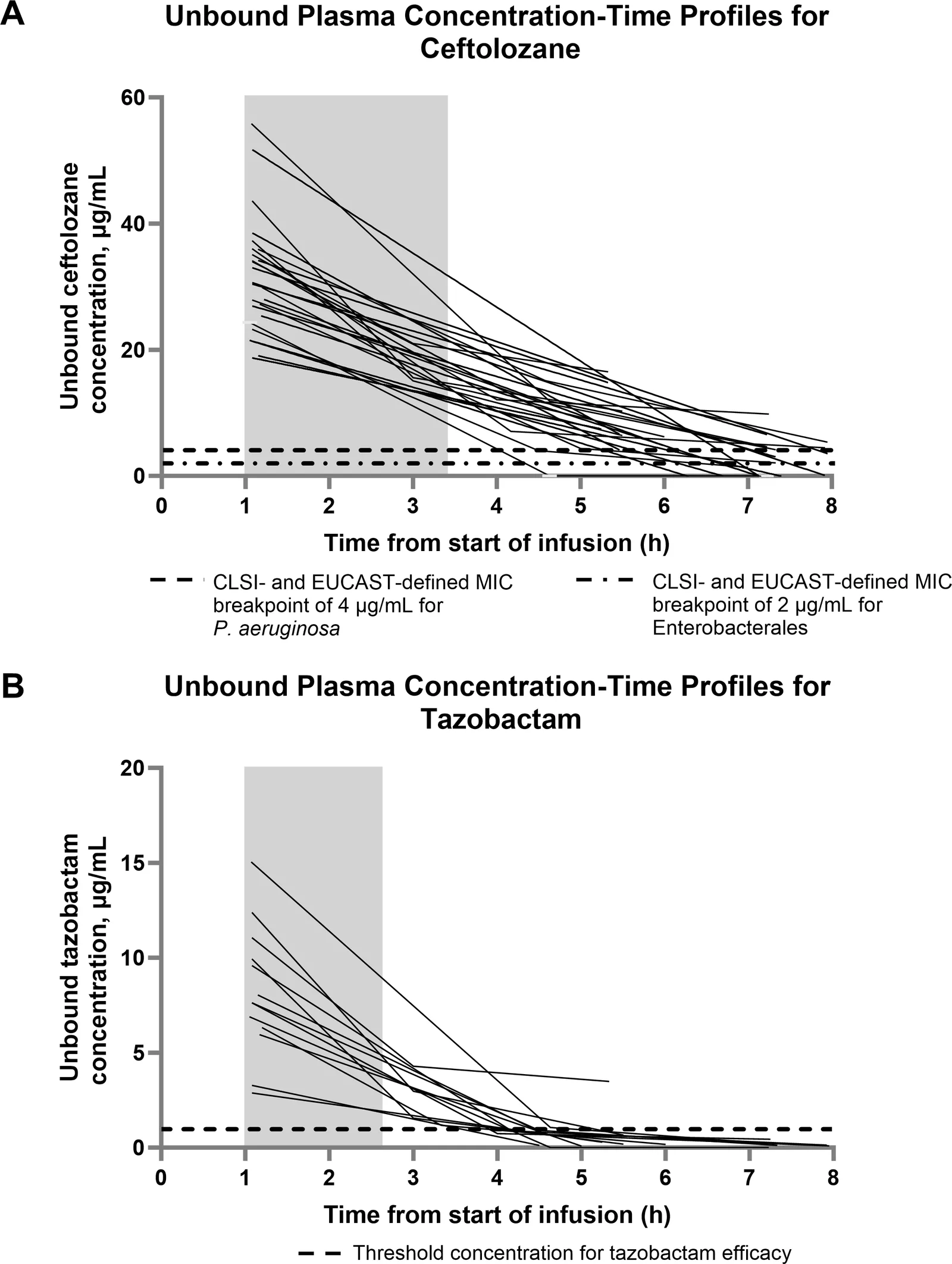
Individual unbound steady-state plasma concentration–time profiles for ceftolozane (**A**) and tazobactam (**B**) in neonates and young infants with pyelonephritis. Shaded areas represent 30% of an 8-h dosing interval (2.4 h) for ceftolozane and 20% of an 8-h dosing interval (1.6 h) for tazobactam. Inset in log scale. CLSI, Clinical and Laboratory Standards Institute; EUCAST, European Committee on Antimicrobial Susceptibility Testing; MIC, minimum inhibitory concentration.

Previously developed population PK models (for ceftolozane and tazobactam) were used to estimate post hoc individual steady-state PK parameter values after IV administration, and these models accounted for renal maturation effect on clearance, which primarily impacts neonates and young infants (32). The steady-state PK parameter values are included in Table 4. Individual-level unbound plasma concentration–time profiles for ceftolozane and tazobactam are displayed against CLSI- and EUCAST-defined MIC breakpoints (30, 31) in Fig. 2. The individual unbound concentration–time profiles for ceftolozane and tazobactam are displayed in Fig. 2. One patient had measurable end-of-infusion PK samples; however, the remaining PK samples were below the limit of quantification (BLQ) for both ceftolozane and tazobactam. BLQ values for this patient were presented as 0 and included in Fig. 2. For one patient, a sample was collected ~5 min after the start of the infusion; as it was the only sample available during the infusion, it was excluded from the figure. In the plots in Fig. 2, unbound plasma concentrations were calculated by converting the measured plasma total ceftolozane and tazobactam concentrations by the unbound fractions (0.79 and 0.70, respectively). MIC values of 2 µg/mL and 4 µg/mL are overlain to indicate CLSI- and EUCAST-defined MIC breakpoints for Enterobacterales and *P. aeruginosa,* respectively. All neonates and young infants met the joint ceftolozane fraction of the dosing interval that plasma concentrations remain above the MIC (30% ƒT > MIC) and tazobactam fraction of the dosing interval that free plasma concentrations remain above a threshold concentration (20% ƒT > C_T_) target, noted with the shaded region. The protocol allowed provisions for the PK samples to be collected at comparable time points on day 3 or after day 3 where patients were presumed to be a steady state in Fig. 2. Over the 8-h dosing period, peak concentrations for ceftolozane ranged from 18.72 to 55.85 µg/mL and for tazobactam, from 2.89 to 15.05 µg/mL.

**TABLE 4:** Summary [mean (SD)] of steady-state ceftolozane and tazobactam plasma PK parameters^*a*^

	AUC_0–8_ (µg*h/mL)	C_eoi_ (µg/mL)	wnCL (L/h)	wnV_SS_ (L)
Ceftolozane	144 (38)	43.1 (12)	0.15 (0.04)	0.53 (0.15)
Tazobactam	44.6 (15)	25.9 (9.6)	0.27 (0.11)	0.33 (0.16)

^*a*^ Values are for neonates and young infants after dosing with ceftolozane 20 mg/kg and tazobactam 10 mg/kg via 1-h infusion administered every 8 h. AUC_0–8_, area under the curve in the dosing interval 0–8 h at steady state; C_eoi_, concentration at the end of infusion; wnCL, weight-normalized elimination clearance; SD, standard deviation; wnV_SS_, weight-normalized steady-state volume of distribution.

## DISCUSSION

Ceftolozane/tazobactam is a treatment option for children with cUTI, including pyelonephritis (22). This subgroup analysis, in which all patients had pyelonephritis at baseline, demonstrates that the safety, efficacy, and PK results in the neonate and young infant population were generally consistent with the overall pediatric population. Also consistent with the overall pediatric population, *E. coli* was the most common baseline qualifying pathogen in this analysis, followed by *K. pneumoniae* (24).

Reported AEs were generally consistent with those of the other pediatric age groups. Thrombocytosis and neutropenia-related AEs were more common in the ceftolozane/tazobactam arm, but all of the AEs had resolved or were resolving at the time of the last study visit, none led to study drug discontinuation, and none were considered serious (24). There was an increased rate of bacteremia in this neonatal population (20%), which is not unexpected owing to the high risk of urosepsis in neonates having pyelonephritis; microbiologic eradication occurred in all of those patients, including those with obstructive uropathy.

Rates of clinical cure and microbiologic eradication at EOT and TOC in the neonate and young infant subgroup were comparable to those of the overall pediatric population for both treatment groups. The pediatric doses utilized in this study targeted ceftolozane and tazobactam plasma exposures (area under the curve in the dosing interval 0–8 h at steady state (AUC) and concentration at the end of infusion [C_eoi_]) in pediatric patients that would not exceed those in adult cUTI, as well as a joint ceftolozane (%ƒT > MIC) and tazobactam (%ƒT > C_T_) target that would achieve at 1-log kill in murine infection models (33). All neonates and young infants included in Fig. 2 met these criteria. The concentration–time profiles for ceftolozane and tazobactam in the pediatric and young infant cohort were broadly similar to those of the overall pediatric population in the pediatric PK and safety study for ceftolozane/tazobactam (23). The population PK analysis demonstrated that there was substantial overlap in the distribution of the ceftolozane and tazobactam exposures of each of the pediatric age groups (including the neonates and young infants) and adults (33).

Increasingly, regulatory frameworks support the conduct of clinical studies in the neonate and young infant populations. However, the inclusion of this age cohort presents challenges that must be taken into consideration when working with this vulnerable population. To enable the enrollment of neonates and young infants in this study, a number of protocol adjustments were required. These included limiting the number of blood draws and urine collections to minimize risk, permitting the use of sterile urine collection bags for young children unable to provide midstream clean-catch urine specimens at follow-up visits, and allowing for a range of weight-based meropenem dosing schemes between 20 and 30 mg/kg. These adjustments removed barriers to enrollment for neonates and young infants with the intent to minimize risks to patients in the study while collecting sufficient data to evaluate safety and efficacy.

This study was not without limitations. The study excluded patients with eGFR <50 mL/min, which is within the normal range for neonates. This exclusion was necessary because, at the time of protocol development and study conduct, dosing for eGFR <50 mL/min had not yet been determined. Despite this exclusion criterion, the study did include three patients who were neonates. The majority of patients had renal clearance rates of eGFR from 50 to 80 mL/min, which is a higher proportion than that reported for the overall pediatric population in the study but is in alignment with previous reports of eGFR rates in the young infant population (24, 34). The study also did not include any preterm babies (born at <37 wk of gestational age).

In conclusion, ceftolozane/tazobactam had a favorable safety profile and achieved high clinical cure and microbiologic eradication rates in neonates and young infants with cUTI.
